# HIF-1*α* Activation Promotes Luteolysis by Enhancing ROS Levels in the Corpus Luteum of Pseudopregnant Rats

**DOI:** 10.1155/2021/1764929

**Published:** 2021-09-01

**Authors:** Zonghao Tang, Jiajie Chen, Zhenghong Zhang, Jingjing Bi, Renfeng Xu, Qingqiang Lin, Zhengchao Wang

**Affiliations:** ^1^Provincial Key Laboratory for Developmental Biology and Neurosciences, Provincial University Key Laboratory of Sport and Health Science, Key Laboratory of Optoelectronic Science and Technology for Medicine of Ministry of Education, College of Life Sciences, Fujian Normal University, Fuzhou 350007, China; ^2^Key Laboratory of Medical Electrophysiology, Ministry of Education, Medical Electrophysiological Key Laboratory of Sichuan Province, Southwest Medical University, Luzhou 646000, China

## Abstract

The increase of oxidative stress is one of the important characteristics of mammalian luteal regression. Previous investigations have revealed the essential role of reactive oxygen species (ROS) in luteal cell death during luteolysis, while it is unknown how ROS is regulated in this process. Considering the decrease of blood flow and increase of PGF_2*α*_ during luteolysis, we hypothesized that the HIF-1*α* pathway may be involved in the regulation of ROS in the luteal cell of the late corpus luteum (CL). Here, by using a pseudopregnant rat model, we showed that the level of both HIF-1*α* and its downstream BNIP3 was increased during luteal regression. Consistently, we observed the increase of autophagy level during luteolysis, which is regulated in a Beclin1-independent manner. Comparing with early (Day 7 of pseudopregnancy) and middle CL (Day 14), the level of ROS was significantly increased in late CL, indicating the contribution of oxidative stress in luteolysis. Inhibition of HIF-1*α* by echinomycin (Ech), a potent HIF-1*α* inhibitor, ameliorated the upregulation of BNIP3 and NIX, as well as the induction of autophagy and the accumulation of ROS in luteal cells on Day 21 of pseudopregnancy. Morphologically, Ech treatment delayed the atrophy of the luteal structure at the late-luteal stage. An in vitro study indicated that inhibition of HIF-1*α* can also attenuate PGF_2*α*_-induced ROS and luteal cell apoptosis. Furthermore, the decrease of cell apoptosis can also be observed by ROS inhibition under PGF_2*α*_ treatment. Taken together, our results indicated that HIF-1*α* signaling is involved in the regression of CL by modulating ROS production via orchestrating autophagy. Inhibition of HIF-1*α* could obviously hamper the apoptosis of luteal cells and the process of luteal regression.

## 1. Introduction

The corpus luteum (CL) is an ephemeral gland in mammalian ovaries evolved from the remains of ovulated follicles, which is responsible for the maintenance of hormonal homeostasis during the menstrual cycle and pregnancy [1]. Normally, the lifespan of CL is mostly dependent on the presence of pregnancy; this period is termed as the luteal phase. The absence of pregnancy is an essential indicator for the initiation of luteal regression and eventually the removal of CL from the ovarian structure at the end of pregnancy whereupon permitting the initiation of the next menstrual cycle [2]. The regression of CL implicates the degradation of the extracellular matrix, loss of blood vessel, and luteal cell death. The attenuation of CL regression disrupts the ovarian cycle and may create a disordered context impairing thereafter follicular development.

Previous investigations have indicated that the increase of ROS is an essential precursor of rat luteal regression [1]. Furthermore, an in vitro study showed that the level of ROS can also be induced by PGF_2*α*_, a luteolytic agent [3, 4]. In humans, H_2_O_2_ treatment is able to generate a luteolytic effect on granulosa-luteal cells [5]. These findings highlighted the important role of ROS in luteal regression. However, the question in terms of how ROS is generated still remains poorly understood. Physiologically, the degeneration of the vascular network is initiated before the comparable change of the CL structure during luteolysis [6]. Furthermore, precedent evidences also indicate PGF_2*α*_ as a HIF-1*α* activator in adipocytes [7], and this effect was also observed in luteal cells [8]. Therefore, it is rational to hypothesize that HIF-1*α* might be an essential causative factor of ROS generation. Indeed, the prodeath role of HIF-1*α* on luteal cells has been evaluated in an in vitro model [9]. The level of HIF-1*α* downstream protein BNIP3, an important factor involved in autophagy regulation in mammalian cells, is also obviously increased under hypoxic conditions in luteal cells [9]. These findings suggest that the dysregulation of autophagy may be involved in ROS production in luteal cells. Consistently, plenty of evidences revealed the involvement of autophagy in luteal regression in diverse species, including rats [10] and humans [11], although the detail of autophagic regulation still remains elusive.

Autophagy is an evolutionarily conserved catabolic mechanism in eukaryotic cells, by which cells can elegantly control the homeostasis of metabolism under stresses so as to inhibit the occurrence of cell dysfunctions. However, autophagy could also promote the apoptosis of cells by excess self-digestions, removal of essential organelles, and autophagosome accumulation [12]. The abnormal induction of autophagy is highly involved in diverse cell diseases, including cancer [13], diabetes [14], and neurodegeneration [15]. In the mammalian ovary, autophagy plays crucial roles in the formation of the primordial pool [16], the transition of oocyte-to-embryo, the development of follicles [17], and the regression of the corpus luteum [10]. However, the evidence is still marginal about how autophagy is involved in luteal regression.

In the present study, we evaluated the expression changes of HIF-1*α* signaling and its effect on oxidative stress during luteal regression by using a pseudopregnant rat model. We treated rats with echinomycin, a potent HIF-1*α* inhibitor, and studied the progress of luteal regression by observing luteal morphologies and expressions of apoptotic proteins during luteal regression, aiming to elucidate the role of HIF-1*α* signaling in autophagy and apoptosis during the luteal regression of pseudopregnant rats.

## 2. Materials and Methods

### 2.1. Animals

Female Sprague-Dawley rats (about 21~23 days) were purchased from Wushi Experimental Animal Supply Co. Ltd. (Fuzhou, China). The animals were maintained under a 14 h : 10 h light-dark schedule with a continuous supply of chow and water. These rats were superovulated by i.p. 30 IU pregnant mare serum gonadotrophin (PMSG; Ningbo Second Hormone Factory, Jiangsu). After 48 h, these rats were then treated with human chorionic gonadotrophin (hCG; i.p. 30 IU, Ningbo Second Hormone Factory, Jiangsu) to induce pseudopregnancy. The rats were executed, and the ovaries were excised for further analysis on Days 7, 14, and 21 after hCG treatment. The experimental protocol was approved by the Institutional Animal Care and Use Committee and the Ethics Committee on Animal Experimentation, Fujian Normal University. All efforts were made to minimize animal discomfort and to reduce the number of animals used.

### 2.2. Immunohistochemistry

The ovaries were fixed in 4% paraformaldehyde. After fixation, the ovaries were embedded in paraffin, and 5 *μ*m sections were cut and mounted on slides. After drying, the sections were dewaxed and rehydrated before antigen retrieval. The sections were then processed for immunohistochemical analysis with anti-LC-3 antibody (1 : 500 dilution, ab48394, Abcam), anti-Beclin1 antibody (1 : 200 dilution, 11306-1-AP, Proteintech), and anti-p62 antibody (1 : 200 dilution, 18420-1-AP). The negative control used serum (Boster Biological Technology, Wuhan) instead of primary antibody. The sections were incubated at 4°C overnight with a primary antibody. The immunoreactivity of the specific protein was visualized by the Elite ABC kit (BioGenex, San Ramon, CA, USA). Then, the sections were counterstained with hematoxylin and mounted with cover slips.

### 2.3. Western Blotting Analysis

The CLs were separated from ovaries under a dissecting microscope with great care. After that, CLs were homogenized by using ice-cold RIPA buffer with supplemented protease inhibitors (protease inhibitor cocktail, Beyotime Institute of Biotechnology, Haimen, China) for protein extraction. Protein concentrations were determined by a Bio-Rad protein assay (Bio-Rad, Hercules, CA, USA) with bovine serum albumin standards. 20 *μ*g protein samples were subjected to SDS-PAGE gel electrophoresis. The nonspecific binding was blocked by 5% skimmed milk, and the membranes were thereafter incubated overnight in the presence of primary antibodies (Supplemental Table 1). After incubation, the membranes were washed with TBST and then incubated in horseradish peroxidase-conjugated goat anti-rabbit or mouse IgG (1 : 1000 dilution, Beyotime Institute of Biotechnology, Haimen, China) for 1 h at room temperature. Eventually, the bands were visualized by using enhanced chemiluminescence star (ECL, Beyotime Institute of Biotechnology, Haimen, China). The bands were quantified using ImageJ 1.49 software (National Institutes of Health, Bethesda, MD, USA).

### 2.4. Quantitative RT-PCR (qRT-PCR)

Total RNA of luteal samples was extracted with TRIzol (Invitrogen, Carlsbad, CA, USA) and then reverse-transcribed into cDNA using a commercial kit (Bio-Rad, Hercules, CA, USA). Real-time PCR was performed with SYBR Super Mix (Bio-Rad, Hercules, CA, USA) in a reaction volume of 20 *μ*l, and the reaction was processed in the ABI StepOne system (Applied Biosystems, Foster City, CA, USA). Primer sequences include BNIP3 (F: 5′-CTC TGC TGA GTG AAG TTC TAC G-3′, R: 5′-AAC ACA AGT GCT GGA TAC TGA TT-3′), NIX (F: 5′-GCA GTG CCA TTG AAC TGT GG-3′, R: 5′-GGA ACC GCA AAT CGA CAT CG-3′), and GAPDH (F: 5′-CGA CCC CTT CAT TGA CCT CAA C-3′, R: 5′-AAG ACG CCA GTA GAC TCC ACG AC-3′) primers. Expression data were normalized to the expression level of GAPDH.

### 2.5. Cell Treatment

To test the effect of HIF-1*α* on ROS biogenesis, we isolated luteal cells from CL on Day 7 of pseudopregnancy according to the methods provided before [18]. To mimic luteal regression, luteal cells were incubated in OptiMEM (Gibco) medium with the presence of PGF_2*α*_ (100 *μ*M, Sigma) or PGF_2*α*_ and 3-MA (2 mM, Sigma) for 24 h.

### 2.6. ROS Detection

For tissular detection, the equal volume of CLs was dissected from the ovaries and was homogenized in lysis buffer (250 mM sucrose, 20 mM HEPES-NaOH, pH 7.5, 10 mM KCl, 1.5 mM MgCl_2_, 1 mM EDTA, 1 mM EGTA, and protease cocktail inhibitor) [19] to collect the supernatant at 10000g, 4°C for 5 min. The mixes of DCFH-DA and supernatant were prepared according to the method provided by the manufacturer (Jiancheng Biotech Institute, Nanjing, China). After that, mixes were incubated in the dark at 37°C for 30 min. The DCF fluorescence was determined by a microplate reader at an excitation wavelength of 488 nm and an emission wavelength of 535 nm.

The level of intracellular ROS was measured by using a commercial kit (Beyotime, Institute of Biotechnology, Haimen, China). Briefly, cells were washed with cold PBS. After that, incubate the cells with DCFH-DA at 37°C for 20 min. Cells were washed with PBS before measurement; the DCF fluorescence of 20000 cells was determined by a microplate reader at an excitation wavelength of 488 nm and an emission wavelength of 535 nm (BioTek Synergy HT).

### 2.7. Statistical Analysis

All data were presented as the mean ± SE. The significant differences within or between groups were evaluated by one-way analysis of variance, followed by Tukey's multiple range test. Statistical analysis was conducted using SPSS version 20 software, and *P* < 0.05 was recognized as a statistically significant difference.

## 3. Results

### 3.1. Autophagy Was Induced in Luteal Cells during Luteolysis in a Beclin1-Independent Manner

**Figure 1:**
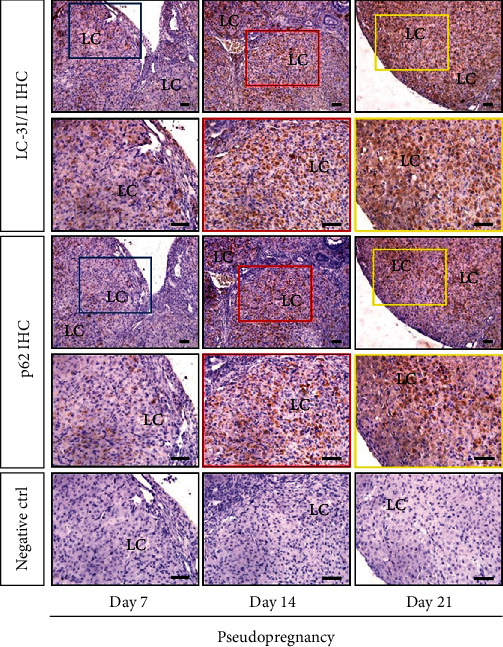
Immunohistochemistry (IHC) of LC-3I/II and p62 on the adjacent section of corpus luteum (CL) from pseudopregnant rats on Days 7, 14, and 21. Negative control (Ctrl) used serum instead of primary antibody. Bar = 100 *μ*m.

**Figure 2:**
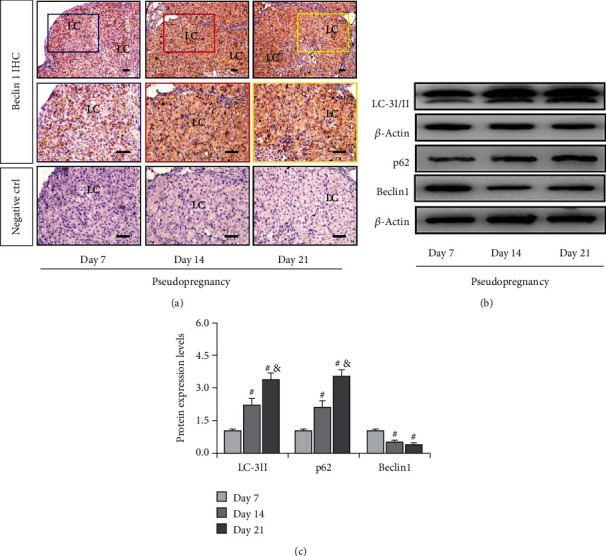
Expressions of LC-3I/II, p62, and Beclin1 in the corpus luteum (CL) from pseudopregnant rats on Days 7, 14, and 21. (a) Immunohistochemistry (IHC) of Beclin1 on the adjacent section of corpus luteum (CL) from pseudopregnant rats on Days 7, 14, and 21. (b) Representative immunoblotting of LC-3I/II, p62, and Beclin1. (c) Densitometric qualification of LC-3I/II, p62, and Beclin1. *P* < 0.05 was considered to indicate a statistically significant difference. ^#^*P* < 0.05, vs. Day 7. ^&^*P* < 0.05, vs. Day 14.

In order to clarify the induction of autophagy during luteal regression, we detected the expression changes of autophagy-related proteins, including the marker protein (LC-3II, Figure 1) of autophagic induction, the marker protein (p62, Figure 1) of autophagosome degradation, and a scaffold protein (Beclin1, Figure 2(a)) involved in autophagic regulation by immunohistochemical staining analysis. These results demonstrated that LC-3II and p62 were expressed in luteal cells and were concomitantly increased during luteal regression (Figure 1), whereas Beclin1 was decreased on Days 14 and 21 of pseudopregnancy (Figure 2(a)). The results of western blotting also verified the increase of LC-3II and p62 (Figure 2(b)) and the decrease of Beclin1 on Days 14 and 21 of pseudopregnancy (Figure 2(b)). These results indicated the decrease of autophagy flux in luteal cells during CL regression, and autophagy is regulated in a Beclin1-independent manner.

### 3.2. The Increase of Cell Apoptosis Is Associated with Oxidative Stress and Skewed Mitochondrial Homeostasis

**Figure 3:**
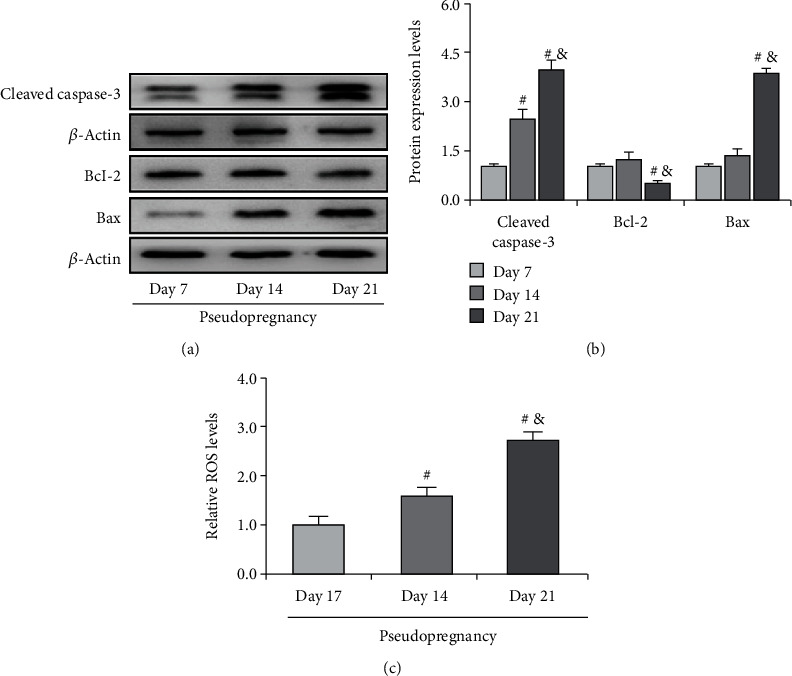
Expressions of cleaved caspase-3, Bcl-2, and Bax in the corpus luteum (CL) from pseudopregnant rats on Days 7, 14, and 21. (a) Representative immunoblotting of cleaved caspase-3, Bcl-2, and Bax. (b) Densitometric qualification of cleaved caspase-3, Bcl-2, and Bax. (c) Relative ROS level in CLs of pseudopregnant rats. *P* < 0.05 was considered to indicate a statistically significant difference. ^#^*P* < 0.05, vs. Day 7. ^&^*P* < 0.05, vs. Day 14.

As we have known that apoptosis was induced in luteal cells during regression, the present study thus examined the expressions of apoptosis-related proteins and found a significant increase of cleaved caspase-3 and Bax on Day 21 (Figures 3(a) and 3(b)) and an obvious decrease of Bcl-2 on Day 21 of pseudopregnancy (Figures 3(a) and 3(b)). Furthermore, we also detected a significant increase in the ROS level in CLs, indicating the occurrence of oxidative stress (Figure 3(c)).

**Figure 4:**
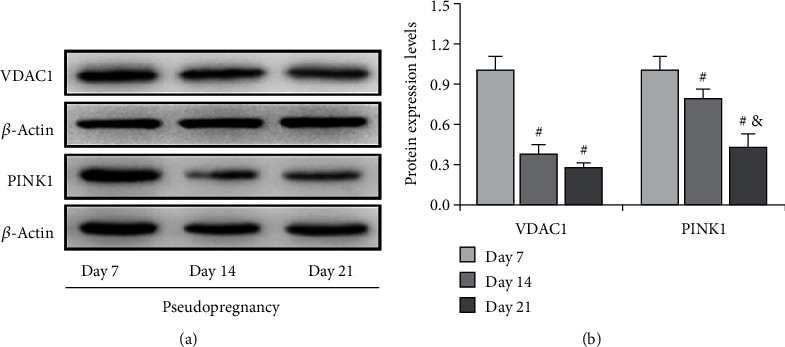
Expressions of VDAC1 and PINK1 in the corpus luteum (CL) from pseudopregnant rats on Days 7, 14, and 21. (a) Representative immunoblotting of VDAC1 and PINK1. (b) Densitometric qualification of VDAC1 and PINK1. Data are presented as the mean ± SE. The significant differences within or between groups were evaluated by one-way analysis of variance, followed by Tukey's multiple range test. *P* < 0.05 was considered to indicate a statistically significant difference. ^#^*P* < 0.05, vs. Day 7. ^&^*P* < 0.05, vs. Day 14.

The elevation of ROS content implicates the disruption of mitochondrial homeostasis. Under stressful conditions, mitophagy is mobilized to degrade mitochondria so as to maintain its homeostasis [20]. We therefore detected the expressions of PINK1 (Figures 4(a) and 4(b)), a regulator of mitophagy induction, and VDAC1 (Figures 4(a) and 4(b)), a mitochondria marker protein. The results demonstrated that both PINK1 and VDAC1 were obviously decreased on Days 14 and 21 of pseudopregnancy (Figures 4(a) and 4(b)), indicating the curtailment of mitochondrial number, and this effect is dominated in a PINK1-independent manner during luteolysis.

### 3.3. HIF-1*α*/BNIP3 Pathway Was Activated during Luteolysis

**Figure 5:**
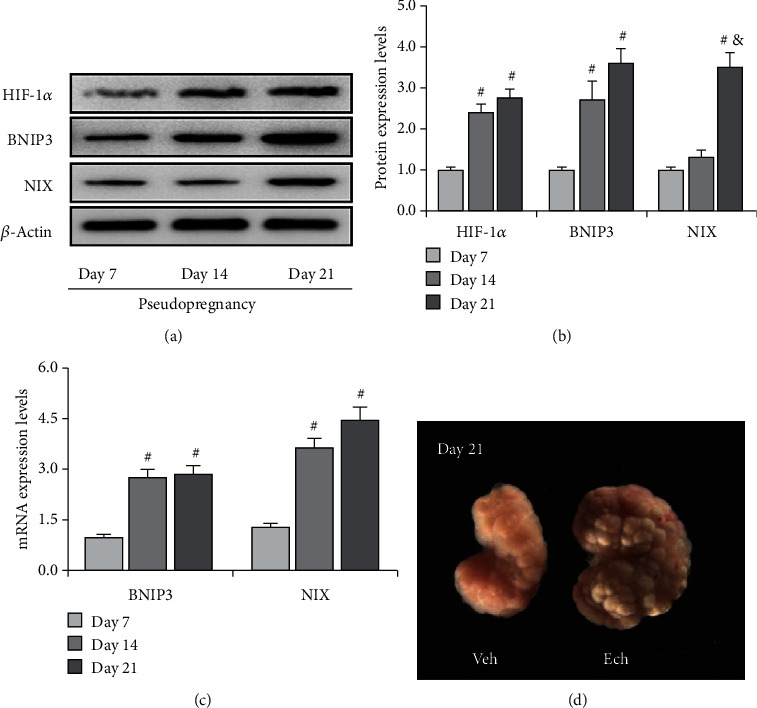
Expressions of HIF-1*α*, BNIP3, and NIX in the corpus luteum (CL) from pseudopregnant rats on Days 7, 14, and 21. (a) Representative immunoblotting of HIF-1*α*, BNIP3, and NIX. (b) Densitometric qualification of HIF-1*α*, BNIP3, and NIX. (c) BNIP3 and NIX mRNA expressions in the corpus luteum (CL) from pseudopregnant rats on Days 7, 14, and 21. (d) Morphology of the ovary on Day 21 from pseudopregnant rats treated with vehicle (Veh) or echinomycin (Ech). *P* < 0.05 was considered to indicate a statistically significant difference. ^#^*P* < 0.05, vs. Day 7. ^&^*P* < 0.05, vs. Day 14.

Previous studies have revealed that hypoxia is involved in the apoptosis of luteal cells *in vitro* [9], implying that HIF-1*α* may participate in apoptosis during luteal regression *in vivo*. Thus, we detected the expression changes of HIF-1*α* signaling and then found an obvious increase of HIF-1*α* on Day 21 of pseudopregnancy (Figures 5(a) and 5(b)), indicating that HIF-1*α* signaling was activated during luteolysis. Furthermore, we examined the expressions of BNIP3 and NIX, two main downstream factors of HIF-1*α* that are involved in autophagy induction [21], during luteal regression. The results showed that the expression of BNIP3 and NIX (Figures 5(a) and 5(b)) is increased in both mRNA and protein levels (Figure 5(c)) on Day 21 of pseudopregnancy, which was consistent with HIF-1*α* expression changes. Interestingly, treatment with HIF-1*α* inhibitor echinomycin (Ech) significantly delayed luteal atrophy during luteolysis (Figure 5(d)), further indicating that HIF-1*α* signaling may play an important role during luteal regression.

### 3.4. Inhibition of HIF-1*α* Signaling Alleviated ROS Production and Cell Apoptosis during Luteolysis

**Figure 6:**
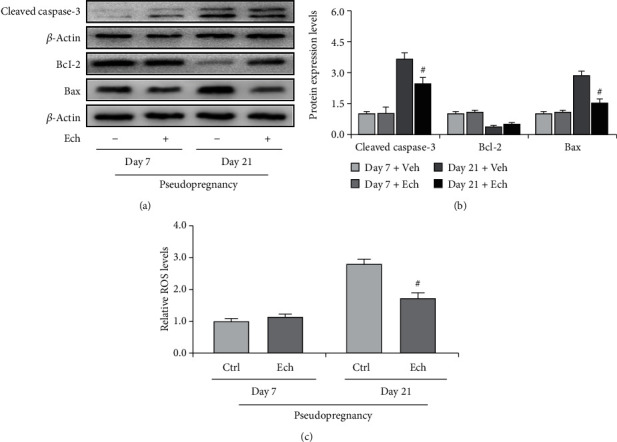
Expressions of cleaved caspase-3, Bcl-2, and Bax in the corpus luteum (CL) on Days 7 and 21 from pseudopregnant rats treated with vehicle (Veh) or echinomycin (Ech). (a) Representative immunoblotting of cleaved caspase-3, Bcl-2, and Bax. (b) Densitometric qualification of cleaved caspase-3, Bcl-2, and Bax. (c) Relative ROS level of CLs with or without Ech treatment on Days 7 and 21 of pseudopregnancy. *P* < 0.05 was considered to indicate a statistically significant difference. ^#^*P* < 0.05, vs. Day 21 without Ech.

Given the fact that Ech retards luteal regression, we thereafter detected the expression of apoptosis-related proteins (Figures 6(a) and 6(b)) and found that Ech treatment obviously inhibited the upregulation of Bax (Figures 6(a) and 6(b)), the decrease of Bcl-2 (Figures 6(a) and 6(b)), and the activation of caspase-3 (Figures 6(a) and 6(b)) on Day 21 of pseudopregnancy. In addition, we also observed a decrease of ROS levels in Ech-treated rats, indicating a positive role of HIF-1*α* on ROS production (Figure 6(c)). The results demonstrated that inhibition of HIF-1*α* signaling could obviously diminish the production of ROS and the activation of caspase-3.

### 3.5. Inhibition of HIF-1*α* Signaling Ameliorated Autophagosome Accumulation

**Figure 7:**
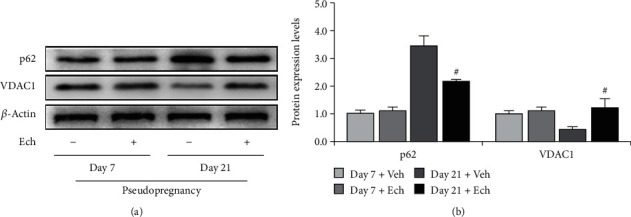
Expressions of LC-3I/II, BNIP3, and NIX in the corpus luteum (CL) on Days 7 and 21 from pseudopregnant rats treated with vehicle (Veh) or echinomycin (Ech). (a) Representative immunoblotting of LC-3I/II, BNIP3, and NIX. (b) Densitometric qualification of LC-3I/II, BNIP3, and NIX. *P* < 0.05 was considered to indicate a statistically significant difference. ^#^*P* < 0.05, vs. Day 21 without Ech.

**Figure 8:**
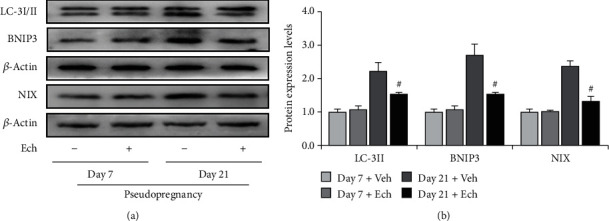
Expressions of p62 and VDAC1 in the corpus luteum (CL) on Days 7 and 21 from pseudopregnant rats treated with vehicle (Veh) or echinomycin (Ech). (a) Representative immunoblotting of p62 and VDAC1. (b) Densitometric qualification of p62 and VDAC1. *P* < 0.05 was considered to indicate a statistically significant difference. ^#^*P* < 0.05, vs. Day 21 without Ech.

To verify the involvement of HIF-1*α* signaling in autophagy induction, we inhibited HIF-1*α* signaling by Ech and then examined the expressions of autophagy-related proteins (Figures 7 and 8) and HIF-1*α* downstream proteins (Figure 7). The results demonstrated that inhibition of HIF-1*α* activity obviously compromised the expressions of BNIP3 and NIX (Figures 7(a) and 7(b)) and also curtailed the levels of LC-3II (Figures 7(a) and 7(b)), which further consolidate that HIF-1*α* signaling participates in autophagy induction during luteolysis. Besides, we also observed the curb of p62 accumulation and the decrease of mitochondrial aggregation in CLs after Ech treatment (Figures 8(a) and 8(b)). Taken together, these findings indicated that HIF-1*α* signaling was involved in the degradation of mitochondria by mediating the upregulation of BNIP3 and NIX during the luteal regression.

### 3.6. Autophagy Is Involved in PGF_2*α*_-Induced ROS Production and Luteal Cell Death

**Figure 9:**
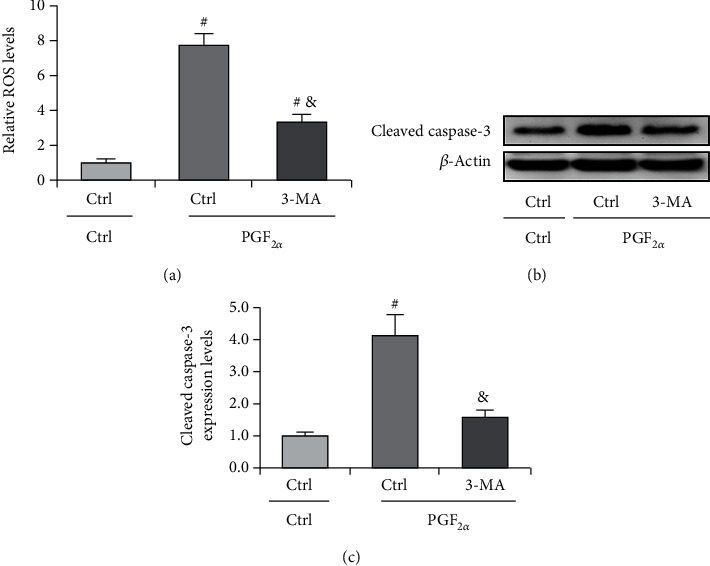
Inhibition of autophagy attenuates PGF_2*α*_-induced ROS production and cell apoptosis. (a) Inhibition of autophagy attenuates ROS production in luteal cells. Cells were treated with PGF_2*α*_ for 24 h with or without 3-MA. (b) Representative immunoblotting and (c) densitometric qualification for the effect of autophagy inhibition on caspase-3 activation. Cells were treated with PGF_2*α*_ for 24 h with or without 3-MA. ^#^*P* < 0.05, vs. PGF_2*α*_.

To test whether dysregulation of autophagy is the causative factor for ROS production, we treated cells with PGF_2*α*_ to mimic luteal regression. Expectedly, we observed the increase of ROS under PGF_2*α*_ treatment. However, inhibition of autophagy by 3-MA obviously abrogated the increase of ROS (Figure 9(a)). This result indicated that increase of autophagy is, at least in part, the upstream of ROS generation during luteal regression. In addition, we also tested the effect of autophagy inhibition on cell apoptosis by detecting the expression of cleaved caspase-3. The results showed that caspase-3 activation is diminished after 3-MA treatment (Figure 9(b)), which is consistent with the trend of ROS generation. These findings suggested that autophagy can promote luteal cell apoptosis partially in an ROS-dependent manner.

## 4. Discussion

The corpus luteum is an important temporary gland in the mammalian ovary, the primary function of which is responsible for the production of progesterone and the maintenance of hormonal homeostasis during the menstrual period and pregnancy [1]. At the end of the menstrual period or the absence of pregnancy, CLs cease to produce progesterone and thereafter begin to regress so as to ultimately eliminate luteal structure from the ovary. Several lines of evidence have established that oxidative stress is engaged in luteal regression in rat [4] and human [5]. Furthermore, these findings demonstrated PGF_2*α*_, a luteal regression inducer, as a critical promoter of ROS production in luteal cells [1]. Particularly, PGF_2*α*_ can also promote luteal cell death by increasing autophagy [10]. The concomitant increase of autophagy level and ROS production rise the possibility that the dysregulation of autophagy is an essential contributor to ROS production and luteal regression. In the present study, we showed that HIF-1*α* enables the upregulation of autophagy levels in luteal cells, which promotes luteal cell death and CL regression by inducing mitochondrial dysregulation and ROS production.

The regression of CL is a multistep biological progress, including functional regression and structural regression [10]. Previously, a wide spectrum of evidence has indicated that apoptosis is the main mechanism that contributes to the atrophy of CLs [11, 22]. Recent investigations also revealed that autophagy is widely involved in the regulation of luteal cell death during luteal regression in cattle [23], human [11], and rat [10]. In mammalian cells, the induction of autophagy plays dual roles in cell survival. The tolerable level of autophagy is required for the resistance of cell death whereas excessive autophagy is deleterious for cell survival. The accumulation of autophagosomes is one of the negative factors induced by autophagy that may disrupt the normal physiology of cells. We here detected the expression of p62, a marker protein indicating autophagosome degradation, Beclin1, an important scaffold protein in the initiation of autophagosomes [24], and LC-3II, a marker protein of autophagy induction. The results revealed that the level of p62 was concomitantly increased with the upregulation of LC-3II, indicating the accumulation of autophagosomes in luteal cells at the late stage of luteal regression. However, we did not observe the upregulation of Beclin1 on Day 14 or 21 of pseudopregnancy, indicating a noncanonical regulation of autophagy in rat CL. Similarly, Gaytan et al. also revealed the curtailment of the Beclin1 level at the late luteal phase of human CL [25], which is consistent with our present finding. Indeed, the induction of Beclin1-independent autophagy was also observed in some cancer cell lines [26–28]. The majority of this noncanonical autophagy regulation was demonstrated to play negative roles in cell survival.

In mammals, the regression of CLs is characterized by the degradation of the capillary network, which induces a hypoxic niche in CLs [1]. Under stressful conditions, cells could mobilize autophagic mechanisms to degrade redundant mitochondria so as to maintain the homeostasis of cellular oxygen consumption under hypoxia [29]. Mechanically, PINK1, BNIP3, and its homologous NIX are recognized as primary mediators involved in the induction of mitophagy in mammalian cells. Among which, BNIP3 mainly undertakes regulatory roles under a hypoxic environment [30–32]. In luteal cells, the expression of BNIP3 is significantly upregulated accompanied by enhanced cell apoptosis under hypoxia, suggesting a positive role in luteal apoptosis [9]. Thus, we examined the change of mitochondrial number during luteal regression and found that the number of mitochondria was decreased on Day 14 and especially Day 21 of pseudopregnancy accompanied by the increase of HIF-1*α*, BNIP3, and NIX. However, we observed a decrease of PINK1 during regression. These findings suggest that HIF-1*α* is involved in mitochondrial loss in late CLs by inducing BNIP3.

The dysregulation of mitochondrial homeostasis is a potential contributor to cell apoptosis, especially by initiating oxidative stress [33]. The atrophy and elimination of CLs implicate massive apoptosis of luteal cells during luteal regression [1], while its underlying mechanism still remains largely unknown. We here revealed that the expressions of apoptosis-related proteins, cleaved caspase-3, and Bax were significantly upregulated during luteolysis, which is consistent with previous reports [34]. Also, we verified the role of HIF-1*α in vivo* by treating pseudopregnant rats with Ech. Expectedly, Ech treatment obviously delayed mitochondrial loss, BNIP3 expression, caspase-3 activation, and the pace of luteal regression when compared with the control. These findings indicated that HIF-1*α* signaling plays a positive role in the apoptosis of luteal cells and the atrophy of CLs during luteolysis. It is noteworthy that autophagy can be regulated by different mechanisms during luteal regression and may also exist divergences between species [23].

Previously, *in vitro* studies have suggested that PGF_2*α*_, an important trigger of mammalian luteal functional regression, is a crucial inducer of oxidative stress and apoptosis in rat luteal cells [1]. Of note, it also exhibits a positive effect on autophagy regulation [10]. In the present study, we demonstrated that autophagy-induced luteal cell apoptosis is partially dependent on oxidative stress, and inhibition of autophagy can ameliorate ROS production and cell apoptosis.

Taken together, in the present study, we found that the HIF-1*α*/BNIP3 signaling pathway is involved in the apoptosis of luteal cells and the regression of CL by inducing oxidative stress.

## Data Availability

The original contributions presented in the study are included in the article/Supplementary Materials; further inquiries can be directed to the corresponding author.

## Supplementary Materials

Supplementary Materials Supplemental Table 1: antibody information for western blotting.
