# Supplementary material for: HIF-1α Activation Promotes Luteolysis by Enhancing ROS Levels in the Corpus Luteum of Pseudopregnant Rats
Source: Oxid Med Cell Longev. 2021 Sep 1;2021:1764929. doi: 10.1155/2021/1764929 (PMC8429025; doi:10.1155/2021/1764929)
Supplement: Supplementary Materials — Supplemental Table 1: antibody information for western blotting. [file 1764929.f1.doc]

Journal: Oxidative Medicine and Cellular Longevity, Special Issue on Oxidative Stress and Trauma Repair

**HIF-1α Activation Promotes Luteolysis by Enhancing ROS Levels in Pseudopregnant Corpus Luteum**

Zonghao Tang, Jiajie Chen, Zhenghong Zhang, Jingjing Bi, Renfeng Xu, Qingqiang Lin, Zhengchao Wang

**Supplementary Table 1**

Table S1. Antibody informations for western blotting.

| **Antibody Name** | **Company and City** | **Dilution Degree** |
| --- | --- | --- |
| LC-3I/II | Abcam, Cambridge, MA, USA | 1:1000 |
| Beclin1 | Protein Tech Group, Wuhan, China | 1:2000 |
| p62 | Abcam, Cambridge, MA, USA | 1:1000 |
| HIF-1a | Santa Cruz Biotechnology, Dallas, TX, US | 1:500 |
| BNIP3 | Abcam, Cambridge, MA, USA | 1:1000 |
| VDAC1 | Protein Tech Group, Wuhan, China | 1:500 |
| PINK1 | Affinity Biosciences, Cincinnati, OH USA | 1:1000 |
| NIX | Abcam, Cambridge, MA, USA | 1:1000 |
| Cleaved caspase-3 | Cell Signaling Technology, Boston, MA, USA | 1:1000 |
| Bcl-2 | Protein Tech Group, Wuhan, China | 1:1000 |
| Bax | Protein Tech Group, Wuhan, China | 1:1000 |
| β-actin | Protein Tech Group, Wuhan, China | 1:5000 |
| anti-Mouse IgG | Beyotime Institute of Biotechnology, Haimen, China | 1:5000 |
| anti-Rabbit IgG | Beyotime Institute of Biotechnology, Haimen, China | 1:5000 |
